# Propofol suppresses synaptic responsiveness of somatosensory relay neurons to excitatory input by potentiating GABA_A _receptor chloride channels

**DOI:** 10.1186/1744-8069-1-2

**Published:** 2005-01-14

**Authors:** Shui-Wang Ying, Peter A Goldstein

**Affiliations:** 1 C.V. Starr Laboratory for Molecular Neuropharmacology, Department of Anesthesiology, Weill Medical College of Cornell University, 1300 York Avenue, Room A-1050, New York, NY 10021, USA

## Abstract

Propofol is a widely used intravenous general anesthetic. Propofol-induced unconsciousness in humans is associated with inhibition of thalamic activity evoked by somatosensory stimuli. However, the cellular mechanisms underlying the effects of propofol in thalamic circuits are largely unknown. We investigated the influence of propofol on synaptic responsiveness of thalamocortical relay neurons in the ventrobasal complex (VB) to excitatory input in mouse brain slices, using both current- and voltage-clamp recording techniques. Excitatory responses including EPSP temporal summation and action potential firing were evoked in VB neurons by electrical stimulation of corticothalamic fibers or pharmacological activation of glutamate receptors. Propofol (0.6 – 3 μM) suppressed temporal summation and spike firing in a concentration-dependent manner. The thalamocortical suppression was accompanied by a marked decrease in both EPSP amplitude and input resistance, indicating that a shunting mechanism was involved. The propofol-mediated thalamocortical suppression could be blocked by a GABA_A _receptor antagonist or chloride channel blocker, suggesting that postsynaptic GABA_A _receptors in VB neurons were involved in the shunting inhibition. GABA_A _receptor-mediated inhibitory postsynaptic currents (IPSCs) were evoked in VB neurons by electrical stimulation of the reticular thalamic nucleus. Propofol markedly increased amplitude, decay time, and charge transfer of GABA_A _IPSCs. The results demonstrated that shunting inhibition of thalamic somatosensory relay neurons by propofol at clinically relevant concentrations is primarily mediated through the potentiation of the GABA_A _receptor chloride channel-mediated conductance, and such inhibition may contribute to the impaired thalamic responses to sensory stimuli seen during propofol-induced anesthesia.

## Background

General anesthesia consists of five distinct components: analgesia, amnesia, unconsciousness, immobility, and blunted autonomic responsiveness [1,2]. While the spinal cord is considered to be the anatomic substrate for anesthetic-induced immobility in response to noxious stimulation [3,4], the anatomic foundations for the other components are less well established. The thalamus is a key integrative structure for somatosensory transmission [5] and, in particular, ascending nociceptive information processing [6,7].

Excitatory input regulates the functional state of thalamic neurons, and such input is provided by both ascending activating systems in the brain stem and hypothalamus and the descending (corticothalamic) pathway [8]. Corticothalamic axons outnumber thalamocortical axons by ~10-fold [9], and activation of this massive descending input depolarizes thalamic neurons, including thalamocortical relay neurons in the ventrobasal (VB) complex, facilitates relay spike transfer, and/or alters the response mode of thalamic relay neurons [10-18]. Inhibitory control of thalamocortical neurons in rodents is provided exclusively by GABAergic neurons in the reticular thalamic nucleus [8,19], and such control is mediated by disynaptic (cortex to RTN to VB) and monosynaptic (RTN to VB) connections.

Propofol (2-6-di-isopropylphenol) is a widely used intravenous anesthetic with a distinct chemical structure, and is a potent allosteric modulator of GABA_A _receptors [20,21]. Recent clinical findings have revealed possible sites of propofol-elicited anesthetic action in the human brain [22,23]. During propofol-induced unconsciousness in humans, somatosensory-evoked neuronal activity in the cortex and the thalamus is markedly decreased [24,25]. *In vivo *extracellular recordings have also demonstrated that propofol suppresses field potentials in the rat thalamus and cortex, with more prominent effects in the cortex [26]. However, the cortical suppression may reflect anesthetic actions on projection neurons located elsewhere, especially in the thalamus [22,27]. A significant limitation to the *in vivo *data from anesthetized animals is the use of "background anesthesia" (typically induced by urethane, sodium pentobarbital or a ketamine/xylazine combination) for baseline recordings; such "background anesthesia" makes it impossible to interpret the data subsequently obtained with the anesthetic(s) of interest [28].

Propofol modulates GABA-evoked currents in heterologously expressed GABA_A _receptors containing an α1, α2, α4, α5, α6, δ or γ2L subunit [29-37]. Behavioral studies suggest that the β3 subunit is important in mediating propofol-induced unconsciousness and immobility [38], while the β2 subunit may mediate sedation [39]. Propofol potentiation of GABA-evoked currents in heterologously expressed GABA_A _receptors is independent of the β1 subunit [29].

Cumulative data from a number of studies using a variety of techniques (including electrophysiology, gene knockout, immunohistochemistry, immunoprecipitation, and ligand binding) suggest that VB neurons primarily express synaptic α1β2γ2 and α4β2γ2 and extrasynaptic α4β2δ GABA_A _receptors while RTN neurons are likely to preferentially express synaptic α3β3γ2 GABA_A _receptors, with denser GABA receptor expression in VB than in RTN [40-58]. These data further support the hypothesis that the thalamus represents an important anatomic target for propofol.

The thalamus is central to the processing and transfer of nearly all sensory information that ultimately reaches the cortex, with the exception of olfaction, whose signals pass to the cortex without thalamic relay. Clinical observations strongly suggest that thalamic neuronal circuits are important targets for propofol. The effects of propofol at the cellular and synaptic levels in the thalamus are largely unknown, however. Therefore, we investigated the effect of propofol on synaptic integration and action potential firing in response to corticothalamic pathway stimulation in thalamocortical relay neurons in brain slices, using both current- and voltage-clamp recording techniques. The results demonstrated that propofol inhibited VB neurons by potentiating GABA_A_-receptor chloride channel-mediated currents. Preliminary results have been published in abstract form [59]

## Results

**Figure 1:**
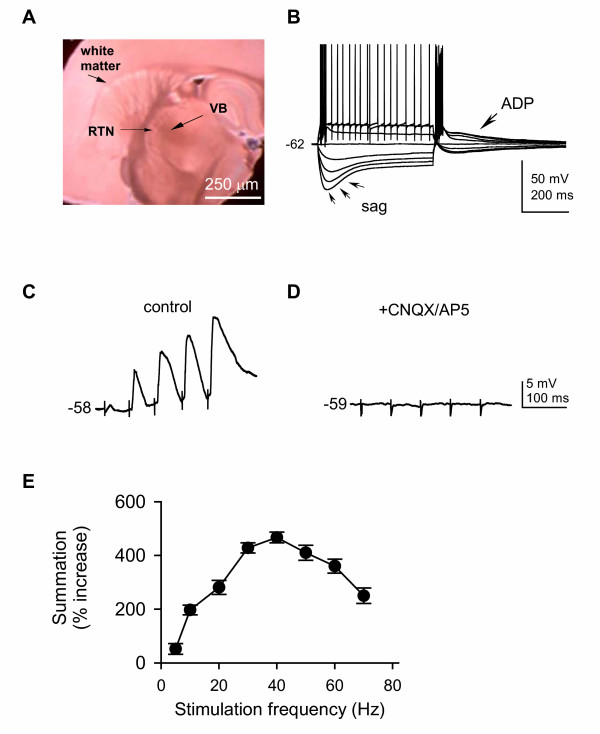
**Synaptic temporal summation in VB relay neurons**. ***A***: a photomicrograph of a live brain slice containing the ventrobasal (VB) complex, reticular thalamic nucleus (RTN), and other regions with which VB has synaptic connections. Thalamocortical slices were cut at 55° (Agmon and Connors, 1991). ***B***: both tonic and rebound burst firing patterns were initiated in a VB neuron with intracellular current pulses (protocol not shown). A distinct membrane voltage response is characterized by a prominent sag and after-depolarization potential (ADP) in response to hyperpolarizing current pulses. The value to the left of the trace indicates membrane potential (mV) here and throughout. ***C***: EPSPs showing temporal summation were evoked by extracellular stimulation of corticothalamic fibers in the white matter (a train of 5 pulses, 33 Hz, 0.15 ms). ***D***: evoked EPSPs could be blocked by CNQX/AP5. ***E***: group data demonstrating that EPSP temporal summation is frequency-dependent (n = 10).

Under low power magnification, the VB was easily discerned in brain slices (Fig. 1A), and with the aid of IR-DIC optics, neurons in VB were readily identified (not shown). VB neurons generally showed a large, slow depolarizing sag and slow after-burst depolarization (ADP, Fig. 1B) in response to deep hyperpolarizing current steps [17]. Biocytin labeling in a subset of cells identified the recording site relative to anatomical landmarks (not shown).

### Propofol suppresses temporal summation in VB neurons

The responses of VB neurons *in vivo *to somatosensory stimuli depend on the state of arousal, and the functional state is linked to neuronal depolarization levels that can be regulated by corticothalamic excitatory (CT) input [8]. CT excitatory synapses in VB exhibit prominent frequency-dependent summation [13,16,60-62]. We therefore examined the effect of propofol on CT-evoked temporal summation in VB neurons. Repetitive stimulation (33 Hz, 5 pulses) of the white matter gave rise to incremental excitatory postsynaptic potentials (EPSPs) that showed summation without apparent inhibitory postsynaptic potentials (IPSPs) at membrane potentials of -58 to -54 mV (Fig. 1C), identical to those seen by others [60,63]. CT-evoked EPSPs had an average latency of 3.2 ± 0.3 ms (n = 55). In some cases, summation could lead to spike firing at the 5^th ^EPSP (not shown); for ease of comparison, we only analyzed those responses without spikes. CT-evoked EPSPs could be abolished by CNQX and AP5 (Fig. 1D), consistent with frequency-dependent facilitation mediated by both NMDA and non-NMDA receptors [64]. The degree of CT EPSP summation increased with increasing stimulation frequency, with a dramatic increase (400–480%) in summation at 33–40 Hz (Fig. 1E).

**Figure 2:**
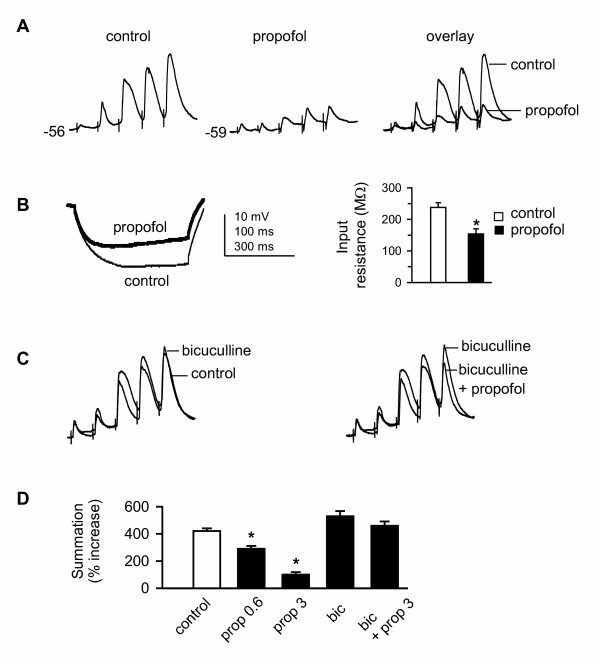
**Propofol suppresses temporal summation via a shunting mechanism**. ***A***: (*left*) control EPSP summation was recorded as described for Fig. 1C. Bath application of propofol (3 μM, 20 min) markedly decreased EPSP amplitude and summation (*middle*). Overlay (*right*) comparing EPSPs in the absence and presence of propofol. ***B***: overlay (*left*) comparing voltage responses elicited by a hyperpolarizing current pulse (-60 pA, 500 ms, not shown) in the absence (control, thin line) and presence of propofol (thick line) in the same neuron as in ***A***. Note that propofol decreased apparent input resistance. Bar graph (*right*) showing that propofol significantly decreases input resistance. *: P < 0.05, n = 15. Time scales: 100 ms for ***A ***and 300 ms for ***B***. ***C***: control EPSPs were recorded from a different VB neuron. Overlay (*left*) comparing EPSPs in the absence (control) and presence of the GABA_A _antagonist bicuculline (10 μM). Overlay (*right*) comparing EPSPs in the presence of bicuculline alone and bicuculline + propofol (3 μM). In the presence of bicuculline, propofol had no significant effect on temporal summation, indicating propofol-elicited shunting inhibition was mediated by GABA_A _receptors in VB neurons. ***D***: group data showing that propofol decreases temporal summation through potentiation of GABA_A _receptors. *: P < 0.05, one-way ANOVA, *vs*. control. n = 15/each group. prop = propofol (0.6, 0.3 μM), bic = bicuculline (10 μM).

We next examined the effect of propofol on temporal summation of EPSPs evoked at 33 Hz. Bath application of propofol (3 μM) hyperpolarized the MP by 3–5 mV, and markedly suppressed the magnitude of CT EPSP summation (102.4 ± 18.5%) in 15 relay neurons tested (Fig. 2A), and the degree to which propofol decreased summation was concentration-dependent (Fig. 2D). Propofol also decreased EPSP amplitude by 30–75% and input resistance by 35.2 ± 4.1% (P < 0.05, Fig. 2B), consistent with shunting inhibition. In addition, small IPSPs were evoked during propofol application (Fig. 2, *middle*).

To determine whether propofol-modulation of GABA_A _receptors contributed to the decrease in summation and input resistance, the experiments were repeated in the presence of GABA_A _receptor chloride channel blockade. In another group of cells (n = 10), bicuculline (10 μM) alone had no significant effect on CT EPSP summation evoked by the same repetitive stimulation as above (Fig. 2C left panel), and a similar response was observed for picrotoxin (100 μM, not shown). Our data were nearly identical to those observed by others [60]. The failure of either GABA_A_-R blocker to markedly increase temporal summation was likely due to the fact that disynaptic inhibition generated within the cortex-RTN-VB circuit was markedly reduced during repetitive stimulation, which resulted in excitatory response only [60]. Propofol, when co-applied with bicuculline, failed to decrease temporal summation (Fig. 2C–D), indicating that GABA_A _receptor-mediated shunting inhibition was involved. The effects of propofol on integrative properties of synaptic responses are summarized in Table 1.

**Table 1:** Effects of propofol on integrative properties of evoked EPSPs in thalamic VB neurons.

	Slope (mV/ms)	1/2 width (ms)	Decay time (ms)
control	1.8 ± 0.4	14.5 ± 2.6	13.2 ± 2.8
propofol	1.6 ± 0.4	6.3 ± 0.4**	16.3 ± 3.2*
+ bicuculline	1.9 ± 0.5	13.5 ± 2.1	12.2 ± 2.4

EPSPs are evoked by extracellular stimulation of corticothalamic fibers (a train of 5 pulses, 0.15 ms, 33 Hz). Data are derived from experiments as described in Fig. 2. Slope (dv/dt) representing depolarization rate is measured from the rising phase (20–80%) of the first EPSP. 1/2 width is measured at 50% of peak amplitude; decay time (20–80%) representing repolarization rate is measured from the falling phase of the fifth EPSP. *, P < 0.05. **, P < 0.001, *vs*. control.

### Propofol decreases spike firing evoked at corticothalamic synapses in VB

**Figure 3:**
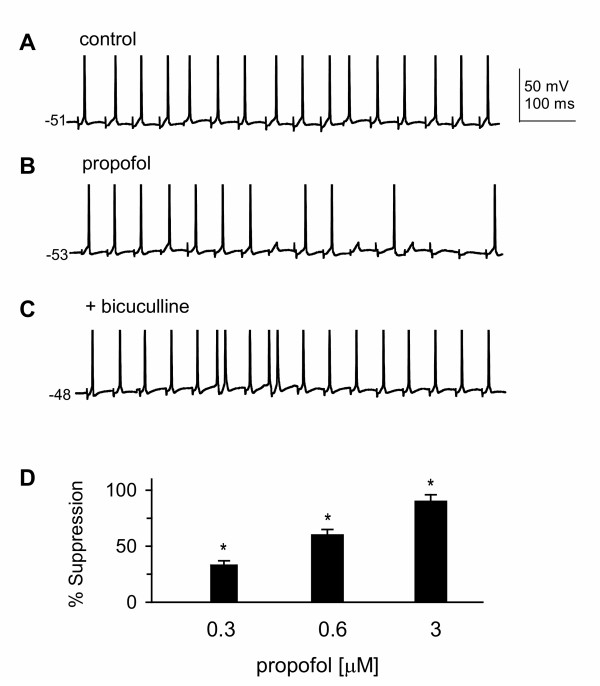
**Propofol decreases corticothalamic-evoked spike firing in VB neurons**. ***A***: action potentials were evoked by corticothalamic (CT) stimulation in a VB neuron under control conditions. ***B***: bath-application of propofol (0.6 μM) suppressed spike generation, and this suppression could be blocked by 10 μM bicuculline (***C***). Propofol was applied 20 min before the trace in (**B**) was recorded. ***D***: bar graph indicates that propofol suppressed successful synaptic transmission (% suppression) at CT synapses in VB in a concentration-dependent manner. *, P < 0.05, one-way ANOVA, *vs*. control, n = 15.

As shown above, propofol suppressed glutamatergic excitatory subthreshold responses (EPSPs) *via *modulation of GABA_A _receptor chloride channels. Such suppression might reduce spike generation in response to excitatory synaptic input. We therefore investigated whether propofol could affect corticothalamic-evoked spike firing. VB neurons were held at depolarized membrane potentials (-51 to -48 mV); stimulation (10–15 Hz) of CT fibers evoked single spike firing in 25 neurons tested (Fig. 3A) with an average latency of 4.2 ± 1.1 ms, and evoked spikes could be blocked by TTX (not shown). These observations are consistent with other reports [13,60]. Bath application of propofol significantly decreased the number of evoked spikes in a concentration-dependent manner (Fig. 3B and 3D). The effect of propofol could be completely blocked by bicuculline (Fig. 3C), confirming the involvement of GABA_A _receptors.

### Propofol inhibits tonic firing by increasing GABAergic input

**Figure 4:**
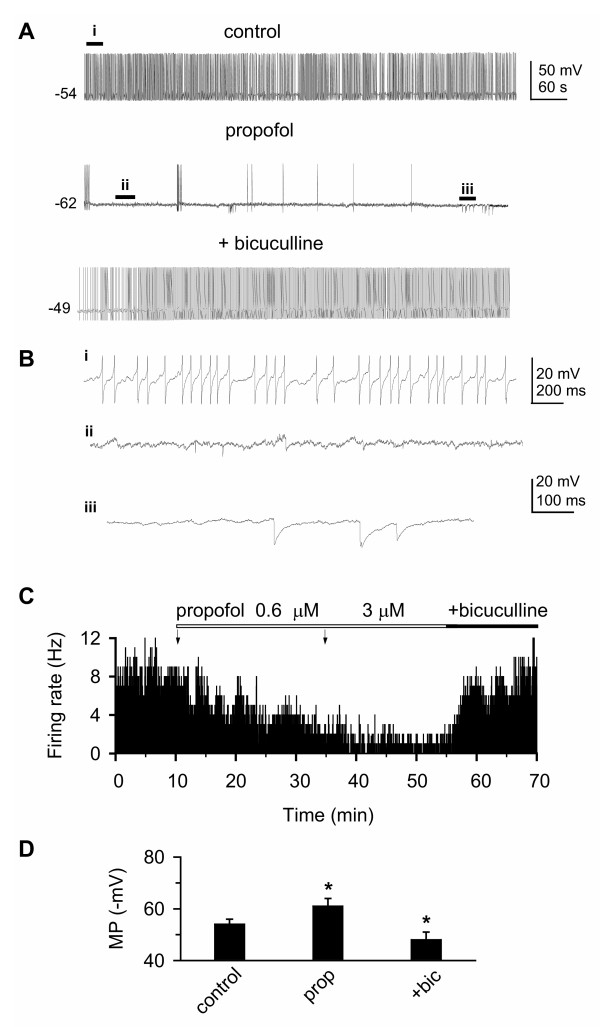
**Propofol suppresses *trans*-ACPD-evoked tonic spike firing in VB neurons**. ***A***: bath application of the metabotropic glutamate receptor agonist *trans*-ACPD (100 μM), which can mimic corticothalamic excitatory transmission [63], induced a sustained, tonic spike firing pattern (*top*). Addition of propofol (3 μM) depressed firing (*middle*), accompanied by spontaneous IPSPs (sIPSPs). The suppression could be blocked by bicuculline (10 μM; *bottom*). ***B***: segments marked with "*i*, *ii*, *iii*" in ***A ***are expanded to view sIPSPs. Spikes are truncated for clarity. Note that few sIPSPs are seen prior to propofol application (*i*), small sIPSPs (< 6.5 mV, *ii*) and large sIPSPs (6.5 – 16.5 mV, *iii*) are observed following propofol application. Scale: 20 mV, 200 ms for *i*, 20 mV, 100 ms for *ii *and *iii*. ***C***: time course histogram for group data (n = 8) showing propofol suppression of *trans*-ACPD-induced tonic spike firing rate. Arrows indicate the onset and duration of propofol application at given concentrations. The suppression of propofol could be blocked by bicuculline. SE bars (0.5 – 2.2) are omitted for clarity. D: bar graph of cumulative data indicates that propofol (prop) hyperpolarized the membrane potential (MP) and this effect was reversed during addition of bicuculline (+bic). *: P < 0.01, one way ANOVA with Tukey test, propofol vs. control, propofol vs. propofol + bicuculline. n = 8 – 12/each.

Thalamocortical neurons *in vivo *fire tonic single spikes during the waking state or in response to excitatory synaptic stimuli [8]. In brain slices, however, these neurons generally do not fire spontaneously; a sustained single spike firing pattern can be induced by pharmacological activation of metabotropic glutamate receptors (mGluRs) with *trans*-ACPD [63]. Since propofol at concentrations less than 10 μM does not affect glutamatergic transmission [65-70], we tested whether propofol could inhibit *trans*-ACPD-induced spike firing through GABAergic mechanisms. Bath application of *trans*-ACPD (100 μM) gradually depolarized the membrane of VB relay neurons by 14 ± 4 mV (n = 12) from resting membrane potentials, and resulted in the generation of sustained, tonic spike firing (Fig. 4A*top*), an effect similar to that seen in the dorsal lateral geniculate nucleus of the thalamus [63]. After a stable tonic, firing pattern was obtained during ACPD application (> 10 min), propofol was added (Fig. 4A*middle*). Propofol markedly decreased both firing rate, and apparent input resistance (P < 0.05, n = 8, one-way ANOVA, vs. control, traces for measuring input resistance not shown).

Although action potential firing in VB neurons was inhibited, RTN neurons continued to fire spikes, as evidenced by the presence of spontaneous IPSPs (sIPSPs, Fig. 4B). The sIPSPs appeared to result from propofol-induced activation of RTN neurons, rather than direct excitation of RTN by *trans*-ACPD, as few, if any, sIPSPs occurred prior to propofol application. The propofol-induced suppression could be blocked by subsequent addition of bicuculline (Fig. 4A*bottom*), indicating that the suppression of VB neuron spike firing was mediated by GABA_A _receptors. Group data demonstrated that propofol significantly suppressed tonic spike firing initiated by pharmacological activation of mGluRs in a concentration-dependant manner (Fig. 4C, P < 0.001, n = 8, one-way ANOVA, vs. control). Propofol hyperpolarized the membrane potential, and the hyperpolarization persisted throughout propofol application (Fig. 4A, *middle*). This effect could be reversed by addition of bicuculline (Fig. 4A, *bottom*) or picrotoxin (not shown). Group data (Fig. 4D) demonstrated that propofol significantly hyperpolarized the membrane potential from -53.7 ± 1.6 to -65 ± 2.9 mV (P < 0.01, same data set as above), strongly suggesting that propofol likely potentiated the tonic GABA_A _receptor-mediated current.

In another subgroup of cells (n = 4, not illustrated), bicuculline alone was added after ACPD induced the tonic firing rate, followed by co-application of propofol (3 μM) and bicuculline. Bicuculline alone increased the firing rate by 16.8 ± 3.2% and the addition of propofol failed to suppress the firing rate.

### Propofol enhances GABA_A _receptor-mediated currents

**Figure 5:**
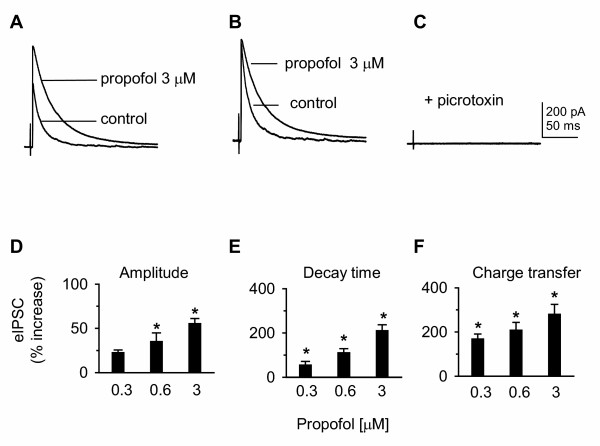
**Propofol-potentiated IPSCs are mediated by GABA_A _receptor chloride channels**. ***A***: GABA_A _IPSCs were evoked in a VB relay neuron in a horizontal slice by RTN stimulation (50 μA, 180 μs, every 15 s) in the presence of the GABA_B _antagonist 2-OH saclofen (100 μM). The membrane potential was clamped at -60 mV. Synaptic currents were potentiated by propofol (3 μM, 10 min). Overlay showing current amplitude in the absence (control) and presence of propofol. Each trace is an average of 10 sweeps. ***B***: normalized traces showing that propofol prolonged the decay time of IPSCs. ***C***: propofol-potentiated eIPSCs could be abolished by picrotoxin (100 μM). ***D-F***: bar graphs of pooled data indicate that propofol increased eIPSC amplitude, decay time, and charge transfer in a concentration-dependent manner. *: P < 0.05, one-way ANOVA, *vs*. control, n = 20.

Our data strongly suggested that propofol-elicited inhibition of VB neurons was primarily mediated through potentiation of the GABA_A _receptor Cl^- ^channel current; this possibility was directly investigated using voltage-clamp recordings. Electrical stimulation of RTN evoked fast unitary IPSCs (eIPSCs) in relay neurons (the membrane potential clamped at -60 mV), with an average latency of 2.4 ± 0.8 ms (Fig. 5A). The eIPSC amplitude was 685 ± 28 pA (n = 30), and the two-exponential decay time (10–90%) was 16.2 ± 2.2 ms. Bath application of propofol increased current amplitude and prolonged current decay time (Fig. 5A–B), and the propofol-potentiated current could be completely abolished by picrotoxin (100 μM), indicating that the response was mediated by GABA_A _receptor chloride channels (Fig. 5C). Inhibitory efficacy of IPSCs can be estimated by calculating total Cl^- ^charge transfer [44]. The efficacy of the eIPSC in control was 1.2 ± 0.8 pC, equivalent to 7.2 × 10^6 ^Cl^- ^ion transfer. Group data demonstrated that propofol increased eIPSC amplitude, decay time, and charge transfer in a concentration-dependent manner (Fig. 5D–F).

## Discussion

The present study demonstrated for the first time that the intravenous anesthetic propofol, at clinically relevant concentrations, suppressed corticothalamic-evoked EPSP temporal summation and action potential firing in thalamic somatosensory relay neurons in VB *in vitro*. The importance of corticothalamic excitatory input in the regulation of thalamic information processing and transfer has been stressed by recent evidence from *in vivo *and *in vitro *experiments. For example, activation of corticothalamic input facilitates single spike firing in thalamocortical neurons [13,64], and alters thalamocortical responses to peripheral sensory stimuli [71-73]. *In vivo *studies in humans have shown that propofol, at plasma levels sufficient to produce unconsciousness, suppressed nociceptive [25] and non-nociceptive stimulus-induced increases in thalamic blood flow [24], indicating that thalamic activity was decreased. Our data provide unambiguous support for the hypothesis that propofol disrupts neuronal activity and synaptic transmission in the thalamus.

### The thalamus is an important propofol target-site

Propofol-induced unconsciousness in humans is accompanied by thalamic inhibition of somatosensory-evoked activity [25] suggesting that such inhibition may play an important role in contributing to general anesthesia. Evidence supporting this assumption is the fact that propofol, at clinically relevant concentrations, consistently suppressed firing activity in all thalamic neurons tested here. Neurons in other brain areas, however, are relatively insensitive to propofol even at very high concentrations. For example, propofol potentiated GABA_A _receptor-elicited synaptic responses at 50 – 500 μM in the hippocampus [70,74], enhanced GABA-elicited inhibition at 50 μM in the olfactory cortex [75], and suppressed spike firing at 30 – 100 μM in the locus coeruleus [76]. Therefore, our data demonstrate that the corticothalamic circuit is a highly sensitive target for propofol.

### Significance of propofol-induced suppression of thalamic excitatory responses

The transmission of sensory information through thalamic relay neurons to the cerebral cortex is state-dependent: transmission is reduced during slow wave sleep or drowsiness, and is enhanced during the waking state [8]. These changes in thalamic excitability are linked to depolarization of relay neurons, which is primarily regulated by corticothalamic excitatory input, or feedback [13]. Corticothalamic projection neurons in the cortex fire high-frequency single and burst spikes *in vivo*, and such excitatory input can readily lead to temporal summation in thalamic target neurons [8]. Here, we clearly demonstrated that corticothalamic-evoked temporal summation and action potential firing were markedly suppressed during propofol application.

A sustained, tonic, firing pattern in thalamic neurons is prevalent during the waking state [8,13]; such a firing activity is lacking in brain slices. Thus, the metabotropic glutamate receptor agonist *trans*-ACPD was used as a pharmacological means to mimic this firing pattern [63]. We found that propofol also inhibited ACPD-evoked firing through a shunting mechanism, and cessation of spike firing in VB neurons was companied by the appearance of spontaneous IPSPs (Fig. 4). The occurrence of sIPSPs strongly suggests that propofol potentiated GABAergic inhibitory input from RTN to VB [77]. Coherent thalamocortical activity during the waking state appears to be essential for conscious experience [17,78], and propofol-elicited shunting inhibition may disrupt such neuronal activity, thereby producing the behavioral changes seen during general anesthesia [79].

IPSPs can contribute to the sculpting of excitatory potentials, and thereby modulate synaptic integration [80,81]. Such an effect is consistent with our observation propofol produced a GABA_A _receptor-mediated decrease in temporal summation in VB neurons (Fig. 2). In addition, the decrease in temporal summation in VB neurons in response to corticothalamic stimulation parallels the failure of spike transfer shown in Fig. 3. The failure of spike transfer in VB neurons following propofol application supports the observation that feedback inhibition gates spike transmission in hybrid thalamic circuits [82]. It is unlikely that propofol directly suppressed glutamatergic transmission because propofol, at the concentrations used here (< 10 μM), has no effect on glutamate receptors [65,66] or glutamatergic excitatory transmission [69].

### GABA_A _receptors mediate the effect of propofol in VB neurons

Anesthetic suppression of excitatory responses may be mediated by at least two distinct mechanisms: enhancement of GABAergic transmission and direct suppression of glutamatergic transmission. Our results showed that there was strong evidence for shunting inhibition of synaptic temporal summation (Fig. 2) and *trans*-ACPD-evoked spike firing rate (Fig. 4), as a marked decrease in apparent input resistance was observed during propofol application. In addition, propofol caused a *prolonged *hyperpolarization of the membrane potential while inhibiting ACPD-evoked spike firing (Fig. 4), and the hyperpolarization was reversed by bicuculline or picrotoxin. The data strongly suggested that a tonic GABA_A _receptor current may be involved in mediating the inhibition during propofol application, consistent with previous observations [83,84]. The GABA_A _receptor δ subunit is expressed in VB [57], and likely contributes to an extrasynaptic pentameric receptor with an α4βδ configuration [48,53,54]. Propofol potentiates δ subunit-containing GABA_A _receptors when co-expressed with an α4, but not α6, subunit [30,31]. Thus, the propofol-induced hyperpolarization of the cell membrane observed here is consistent with its potentiation of extrasynaptic GABA_A _receptors.

Our data also provide evidence for propofol potentiation of the GABA_A _receptor chloride channel-mediated phasic currents, as propofol markedly increased picrotoxin-sensitive IPSC amplitude, decay time, and charge transfer (Fig. 5). In addition to a pool of synaptic receptors containing an α1 subunit that mediates fast IPSCs [44-47], α4 subunit-containing receptors accounts for ~30% of the total GABA_A _receptor population in the thalamus [48,49]. α4-containing receptors are recognized by [^3^H]Ro15-4513 [48], indicating the presence of a γ2 subunit, and are expressed synaptically [40,50]. Therefore, the pool of synaptic GABA_A _receptors expressed by VB neurons is heterogeneous, consisting primarily of α1- and α4-subunit containing receptors. Propofol markedly increased IPSC amplitude, suggesting potentiation of synaptic receptors containing either an α1 or α4 subunit [30,35]. These receptors are likely to contain a β2 subunit as this subunit contributes to propofol-induced potentiation of GABA-evoked currents [85]. Finally, propofol-increased IPSC decay time suggested that the γ subunit might be involved, since propofol prolonged the deactivation time in receptors expressed in HEK cells containing a γ2L subunit [31]. These data strongly support the conclusion that propofol caused shunting inhibition by enhancing GABA_A _receptor-mediated chloride conductance in VB neurons through both synaptic and extrasynaptic receptors.

## Conclusions

The GABAergic general anesthetic propofol, at clinically relevant concentrations, markedly suppressed excitability and synaptic responsiveness to corticothalamic activation in thalamocortical relay neurons in VB. Propofol enhancement of postsynaptic GABA_A _receptor-function on VB neurons resulted in shunting inhibition of excitatory input. Recent clinical findings [22,24,25,79,86,87] and *in vivo *electrophysiological evidence [28] have all suggested that thalamocortical circuits may constitute a strategic target for some general anesthetics including propofol. Our results support that hypothesis, and clearly establish the link between propofol-mediated inhibition of corticothalamic activation of VB neurons and propofol-enhanced GABA_A _receptor function.

## Methods

### Brain slice preparation

Experiments were performed in accordance with institutional and federal guidelines. Thalamocortical (TC) slices were prepared as described [88] with a slight modification. Briefly, mice (C57BL/6, P25–55) were anesthetized by halothane and decapitated. The head was immediately submerged in ice-cold carbogenated (95% O_2_/5% CO_2_) slicing solution, and the brain was rapidly dissected out. The rostral portion of the brain was cut at 45° or 55°; the rostral end of the brain block was glued to a homemade platform. Slices (240 or 300 μm) were cut on a microslicer (Leica VT 1000S, Wetzlar, Germany) using a sapphire blade (Leica) to yield smooth-surface slices, gently rinsed once in cold artificial cerebrospinal fluid (ACSF) bath solution, and incubated in carbogenated ACSF at 34°C for 1 hr for recovery and at 24°C for at least another 1 hr before use. For horizontal slices, the brain was sagittally cut into two halves along the midline; 240 μm-thick slices containing both VB and RTN were prepared. Experiments were generally performed on TC slices, except those with RTN stimulation that were carried out in horizontal slices.

### Electrophysiology

Current-clamp recordings were performed at 35°C. Slices were perfused with carbogenated ACSF; neurons were visualized and identified using a Zeiss Axioskop (Jena, Germany) equipped with a 2.5 × objective and 40 × water immersion objective with a 2.4 mm working distance and IR-DIC optics. The resistance of the pipette was 3.5–6 MΩ when filled with internal solution. Tight seal (> 2 GΩ) was achieved by application of a small negative pressure, using a 1 ml-syringe. Access resistance (R_a_) ranged from 10–14 MΩ, and was compensated by up to 60%; data were discarded if Ra > 15 MΩ. Input resistance was measured at a holding membrane potential level close to resting membrane potential (RMP) from the voltage response elicited by a small current pulse (-60 pA). Only neurons that showed a stable RMP negative to -60 mV, action potential (AP) overshoot of > 10 mV and R_i _> 150 MΩ (in current-clamp mode) were selected for study. Although cells so selected generally showed stable data records for up to 240 min, pharmacological tests were completed within 90 min to minimize the variation of responses; only one experiment per slice was performed. Liquid junction potentials (11–12.2 mV) for intracellular and bath solutions were calculated by Junction Potential Calculator (Clampex 8, Axon Instruments, Union City, CA), and corrected online or offline. Membrane voltage was filtered at 5 kHz, membrane current at 2 kHz and then digitized at 10 kHz using an Axopatch 200A amplifier connected to a DigiData 1200 interface (Axon).

### Extracellular electrical stimulation

To stimulate CT fibers, a concentric bipolar tungsten electrode (FHC Inc., Bowdoinham, ME) was placed in either layer VI of the barrel cortex or the white matter in TC slices [60]. Single pulses or train pulses were delivered using a Master-8 pulse generator (A.M.P.I., Jerusalem, Israel) controlled by a PC and a constant current stimulus isolator (World Precision Instruments, Sarasota, FL). Responses were considered monosynaptic if the latency jitter was less than 0.4 ms and their rise times were consistent from trial to trial (3 trials). Latency was calculated from start of stimulus to onset of response. To confirm that the effects of propofol were GABA_A _receptor mediated, responses were blocked by a GABA_A _receptor antagonist (bicuculline 10 μM or gabazine 10 μM) or Cl^- ^channel blocker (picrotoxin 100 μM). To evoke IPSCs, the stimulation electrode was placed in RTN, and synaptic currents were recorded in the presence of the GABA_B _receptor antagonist 2-OH saclofen (100 μM), and in some cases the non-NMDA receptor antagonist CNQX (20 μM) and NMDA receptor antagonist D-AP5 (40 μM) were added. CNQX and D-AP5 were also used to block evoked excitatory postsynaptic potentials (EPSPs) and the Na^+ ^channel blocker tetrodotoxin (500 nM) was used to block evoked action potentials.

### Drug application

Drugs were applied by bath superfusion (unless otherwise noted) for at least 10 min prior to data collection using polytetrafluoroethylene (Teflon^®^) tubing and connectors; solution flow rates were 3 ml/min. Propofol was freshly prepared in DMSO and diluted with ACSF to clinically relevant concentrations (0.3 – 3 μM); the final concentration of DMSO was 0.01%, which had no effects on the cells examined. The concentration range was selected based on the fact that a free aqueous concentration of ~2 μM is required to inhibit a response to a painful stimulus in 50% of test mammalian subjects [89].

### Solutions

Slicing solution contained (in mM): 2.5 KCl, 24 NaHCO_3_, 1.25 NaH_2_PO_4_, 234 sucrose, 11 glucose, 10 MgSO_4_, and 0.5 CaCl_2_. ACSF bath solution contained (in mM): 124 NaCl, 26 NaHCO_3,_2.5 KCl, 1.25 NaH_2_PO_4_, 1.2 MgCl_2_, and 2 CaCl_2 _and 11 glucose. Intracellular solution contained (in mM): 130 K-gluconate, 5 NaCl, 2 MgCl_2_, 10 HEPES, 0.5 EGTA, 2 ATP-K, 0.3 GTP-Na, pH adjusted to 7.25 with KOH. K-gluconate was used because the impermeant ion gluconate does not contribute to anesthetic-induced changes in RMP or I-V relationship [90]. Voltage-clamp recordings of inhibitory postsynaptic currents (IPSCs) were made at 25°C, using a Cs^+^-based internal solution [91]. The bath solution for voltage-clamp contained (in mM): 117 NaCl, 25 NaHCO_3, _3.6 KCl, 1.2 NaH_2_PO_4_, 1.2 MgCl_2_, and 2.5 CaCl_2 _and 11 glucose; osmolarity was adjusted to 300 mOsm with sucrose. All bath solutions were freshly prepared on the same experimental day.

### Intracellular biocytin filling

Neurons from 30 mice were intracellularly filled with biocytin (0.5% in the pipette solution). After recording, slices were fixed for 24–72 hrs in phosphate buffer (PB) solution containing 4% paraformaldehyde, transferred to 20% sucrose solution in 0.1 M PB and re-sectioned to 60 -100 μm. After endogenous peroxidases were blocked with phosphate-buffered 3% H_2_O_2_, the slices were incubated with biotinylated horseradish peroxidase conjugated to avidin (ABC-*Elite*, Vector Labs, Burlingame, CA), washed and incubated with DAB for 15 min. Filled neurons were visualized and reconstructed.

### Chemicals

Compounds from Tocris Cookson (Ellisville, MO) were: (+) bicuculline, picrotoxin, gabazine, 2-OH saclofen, (2S)-3-[[(1S)-1-(3,4-dichlorophenyl) ethyl] amino-2-hydroxypropyl] (phenylmethyl) phosphinic acid (CGP55845), 6-cyano-7-nitroquinoxaline-2, 3-dione (CNQX), D-2-amino-5-phosphopentanoic acid (D-AP5), (±)1-aminocyclopentane-trans-1, 3-dicarboxylic acid (*trans*-ACPD). Tetrodotoxin (TTX) was from Alomone Labs (Jerusalem, Israel), and propofol was from Aldrich (Milwaukee, WI).

### Data and statistical analysis

Membrane voltages and currents were analyzed using both Clampfit 9.0 and MiniAnalysis 6 (Synaptosoft, Decatur, GA). To analyze temporal summation containing five responses, the peak of the first and fifth responses were measured from baseline and expressed as ΔV_1 _and ΔV_5_, respectively; responses were calculated as: % increase = [(ΔV_5 _/ ΔV_1_) - 1] × 100. Temporal summation was defined as % increase in depolarization occurring at the soma during a train [92]. Statistical analyses were performed with Sigmastat V 3.0 (SPSS, Chicago, IL) using *t*-test or one-way ANOVA. Data were expressed as means ± SE.

## Declaration of Competing Interests

The author(s) declare that they have no competing interests.

## Authors' contributions

(SWY) – study design, data collection and analysis, manuscript preparation. (PAG) – study design, manuscript preparation.
